# Design, Synthesis, Docking Study and Biological Evaluation of 4-Hydroxy-2*H*-benzo[*e*][1,2]thiazine-3-carboxamide 1,1-dioxide Derivatives as Anti-HIV Agents

**DOI:** 10.22037/ijpr.2020.114153.14695

**Published:** 2021

**Authors:** Ali Imani, Sepehr Soleymani, Rouhollah Vahabpour, Zahra Hajimahdi, Afshin Zarghi

**Affiliations:** a *Department of Medicinal Chemistry, School of Pharmacy, Shahid Beheshti University of Medical Sciences, Tehran, Iran. *; b *Hepatitis and AIDS department, Pasteur Institute of Iran, Tehran, Iran. *; c *Medical Lab Technology Department, School of Allied Medical Sciences, Shahid Beheshti University of Medical Sciences, Tehran, Iran.*

**Keywords:** Design, Synthesis, Benzothiazine-3-carboxamide 1, 1-dioxide, Integrase, Anti-HIV

## Abstract

A novel series of benzothiazine-3-carboxamide 1,1-dioxide derivatives by modifying the piroxicam scaffold was designed, synthesized, and evaluated as anti-HIV agents. The 1,2-benzothiazine-3-carboxamide 1,1-dioxide scaffold consists of hydroxy and carboxamide groups as a chelating motif to form an interaction with Mg^2+^ ions within the integrase active site as a target. Most of the compounds displayed encouraging anti-HIV activity in a cell-based assay. Among them, compounds **13d**, **13l** and **13m** were the most potent with EC_50_ values ranging from 20-25 µM and SI > 26. Docking study of compounds in integrase active site proposed that the mechanism of action of compounds might be through Mg^2+^ chelation within integrase active site. The lack of severe cytotoxicity and favorable anti-HIV activity of benzothiazine-3-carboxamide 1,1-dioxide derivatives support further modifications to improve the potency.

## Introduction

HIV infection and AIDS remains a major challenge to human health and the economy without a successful cure or a viable vaccine. Since its emergence in the early 1980s, HIV has claimed millions of lives globally (1). During the last decades, the introduction of combination antiretroviral therapy (cART) has significantly reduced the morbidity and mortality of patients with HIV-1 infection (2-5). However, the rapid emergence of multi-drug resistance to current therapeutics and toxicity and patient compliance, has hampered the treatment of viral infection (6). Thus, the search for the development of new antiviral agents remains an essential need.

Integrase (IN) is a critical enzyme in the life cycle of HIV without any human homologue enzyme; thus, integrase inhibitors potentially have minimum adverse effects. So, the integrase enzyme has been identified as an interesting and validated target for drug design against HIV (7-10). From the clinical point of view, the inclusion of an IN inhibitor to an antiretroviral therapy regimen improves the efficacy of therapy by potential synergism, without raising extra toxicity (11), and the use of integrase inhibitors will diminish rates of transmitted drug resistance. Therefore, it is recommended that integrase inhibitors be available for first-line therapy of HIV infection (12). IN is a viral enzyme responsible for the insertion of viral DNA into human DNA. Besides, IN also has important roles in reverse transcription of the viral RNA genome (13). IN catalyzes the integration process via two separate steps. At the first step referred to 3’-processing (3’-P), two base pair nucleotides from the 3′-ends of the reverse-transcribed viral DNA were removed. The second reaction termed strand transfer (ST) involves the joining of the 3′-processed viral DNA into the host DNA (14). The integrated viral DNA is transcribed to RNA and then translated into viral proteins. Integration preferentially occurs at certain regions of human DNA (15). Structurally, Integrase is a 32-KDa metalloenzyme consisting of 288 amino acids. IN is active in its multimer forms, dimeric form is involved in catalyzing 3′-P step, and tetrameric form is essential for performing the ST reaction (15). Each monomer consists of three main domains. The N-terminal domain (amino acids 1-50) has a four conserved HHCC (His12, His16, Cys40 and Cys43) zinc-finger region believed to have a function in multimerization of enzyme (16). The catalytic core domain (residues 50-212) and the C-terminal (residues 213-288) involve in non-specific DNA-binding (17). The catalytic core domain (CCD) contains two divalent Mg^2+^ ions in coordination with 3 highly conserved acidic residues (Asp64, Asp116 and Glu152) (18). Divalent metal ions play a key role in enzyme function, and chelating these metal ions by a ligand can prevent enzyme ligation function (19). The chelating ligand forms a ligand–Mg^2+^–IN complex which would sequentially act as a barrier to the formation state of the IN–DNA complex by competing with the target DNA substrate (20).

**Figure 1:**
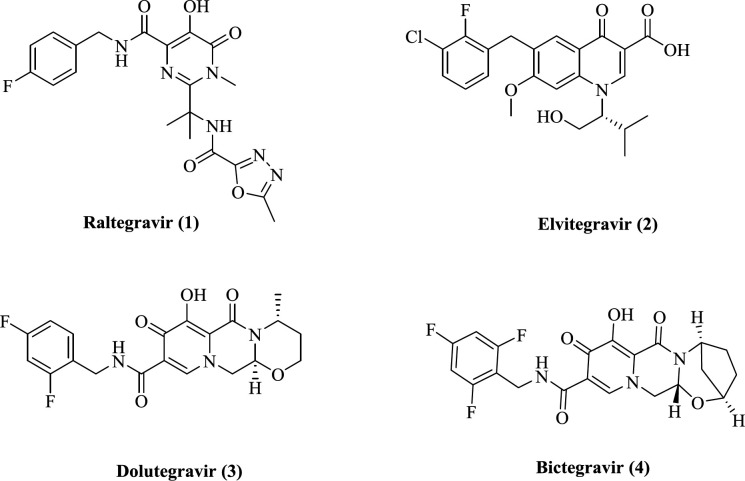
Structure of FDA-approved IN inhibitors (Raltegravir **1**, Elvitegravir **2**, Dolutegravir **3**, Bictegravir** 4**)

A large number of compounds with the ability to chelate metals have been identified and reported (21). This area’s research has led to the introduction of four FDA-approved IN inhibitors, raltegravir, 1, elvitegravir, 2, dolutegravir, 3, and bictegravir** 4** (Figure 1) (22-25). They selectively inhibit ST reaction and known as INSTIs. All INSTIs share a planar chelating group that interacts with Mg^2+^ ions and an aromatic group occupying a hydrophobic region. A flexible linker connects these two groups. Various structural classes of IN inhibitors such as diketo acids, naphthyridine carboxamides, pyrroloquinoline, dihydroxypyrimidine carboxamides, azaindole hydroxamic acids, quinolone-3-carboxylic acids, carbamoyl pyridines have been identified so far (26). However, their cytotoxicity, low potency, and viral resistance hindered further development of such structures (27, 28). So, the discovery of a novel and safe scaffold with sufficient efficacy is of great importance. 

**Figure 2:**
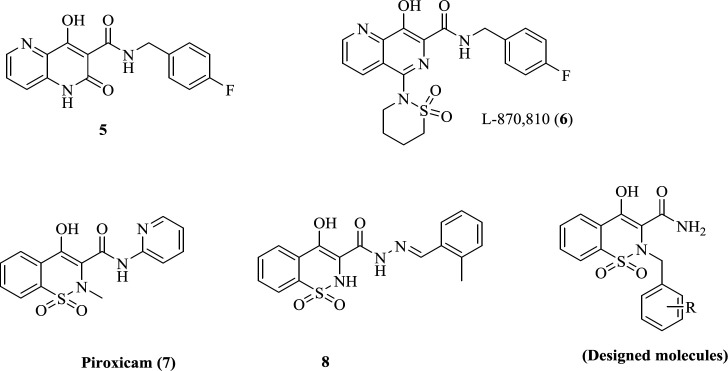
Structure of IN inhibitors and designed molecules

We recently reported piroxicam (**7**) and piroxicam analogs (exemplified by compound **8**) as potential HIV-1 integrase inhibitors with promising anti-HIV-1 activity (Figure 2). 

The main advantage of piroxicam is its highly safe profile, allowing the development of many compounds to be designed on this core. Moreover, it has been reported in several studies that piroxicam can chelate numerous metals such as Mn^2+^, Fe^3+^, Fe^2+^, Co^2+^, Ni^2+^, Cu^2+^, Zn^2+^, Pb^2+^ (29-32), suggesting high potential for chelating other metals like the Mg^2+^ within IN active site. Thus, the main core of piroxicam, 1,2-benzothiazine-3-carboxamide 1,1-dioxide ring, was established as an attractive platform for further optimization in this study. The 1,2-benzothiazine-3-carboxamide 1,1-dioxide scaffold consists of hydroxy and carboxamide groups as a chelating motif to form metal-binding interactions with Mg^2+^ ions. This pattern of chelating motif has also been employed in some well-known potent IN inhibitors such as compound **5** and L-870,810 (**6**) (33) (Figure 2). In designing the current series of piroxicam analogs, the hydrophobic group confirmed to deliver improved potency was shifted from C-3 amide to N-2 position to explore its effect on anti-HIV activity. We report herein the synthesis and anti-HIV activity of 1,2-benzothiazine-3-carboxamide 1,1-dioxide derivatives featuring different hydrophobic groups at the N-2 position. To understand the mode of inhibition, molecular docking studies of these compounds to integrase protein were carried out. 

## Experimental 

*General*

All chemicals and solvents used in this study were purchased from Merck AG and Aldrich Chemical. Melting points were with a Thomas-Hoover capillary apparatus. Infrared spectra were acquired using a Perkin Elmer Model 1420 spectrometer. A Bruker FT-500 MHz instrument (Brucker Biosciences, USA) was used to acquire ^1^H-NMR spectra with TMS as an internal standard. Chloroform-D and DMSO-D_6_ were used as solvents. Coupling constant (*J*) values are estimated in hertz (Hz), and spin multiples are given as s (singlet), d (double), t (triplet), q (quartet), m (multiplet), and br (broad). The mass spectral measurements were performed on a 6410Agilent LCMS triple quadrupole mass spectrometer (LCMS) with an electrospray ionization (ESI) interface. C, H and N elemental analyses were performed on a Costech 4010 elemental analyzer. Microanalyses, determined for C and H, were within ± 0.4% of theoretical values.

*Synthesis of ethyl 2-(1,1-dioxido-3-oxobenzo[d]isothiazol-2(3H)-yl)acetate (* ***10*** *)*

Sodium saccharin (**9**) (30 g, 122 mmol) and ethyl chloroacetate (11.5 mL, 130 mmol) were mixed in DMF (100 mL) and heated to 120 °C for 3 h. The reaction was monitored by TLC. After completion of the reaction, the mixture was cooled down to room temperature. The reaction mixture was poured into ice-cold water. The precipitate was formed, filtered and washed with water. The powder was super dried in a vacuum oven (75 °C, 24 h). Yield: 74%, mp: 123-125 °C ; IR (KBr): ν (cm^-1^) 1750, 1730 (C=O), 1190 (C-O), LC-MS (ESI): *m/z* 292 [M+Na]^+^. 

*Synthesis of ethyl 4-hydroxy-2H-benzo[e][1,2]thiazine-3-carboxylate 1,1-dioxide (* ***11*** *)*

To a 250 mL round bottom flask containing super dried ethanol, sodium (5.750 g, 250 mmol) was added portionwise until all sodium was dissolved. To this solution, compound **10** (27 g, 100 mmol) was added all at once and stirred vigorously under an argon atmosphere. A yellow color was formed quickly, and then the solution turned to an orange slurry. The mixture was allowed to heat under reflux for 2 h. The reaction mixture was cooled and poured into 150 mL of cold HCl (0.5 M). A white precipitate was formed immediately, washed with plenty of water, and then dried under vacuum. Yield: 78%; mp: 138-140 °C, LC-MS (ESI): *m/z* 269 [M+H]^+^, 292 [M+Na]^+^; IR(KBr): ν (cm^-1^) 3166 ( NH), 1663, 1608 (ester carbonyl, enol), 1167 (C-O). 

*Synthesis of 4-hydroxy-2H-benzo[e][1,2]thiazine-3-carboxamide 1,1-dioxide (* ***12*** *)*

Compound **11** (18 g, 67 mmol) was added to a 20% aqueous solution of ammonia. A yellow clear solution was formed after about 10 min. The solution was stirred at room temperature overnight and then acidified by cold HCl (6 M) until a white precipitate was formed. The precipitate was filtered, washed with cold water and crystallized in 50:50 acetone methanol solution. Yield: 85%, mp: 244 °C (decomposed). IR (KBr): ν (cm^-1^) 3490 (NH), 3212, 3253 (NH_2_), 1648 (amide C=O). ^13^C NMR (400 MHz, DMSO-*d6*): 172.04, 155.46, 137.92, 133.08, 132.31, 129.01, 125.96, 122.34, 105.92.

*General procedure for the synthesis of compounds* ***13a-m***

A mixture of compound **12** (3 mmol), the appropriate alkyl or benzyl halides (6 mmol), and solid K2CO3 (4.5 mmol) in dry DMF (10 mL) was stirred at room temperature for 1.5–2 hours and then poured into an ice-water mixture. The precipitate was collected by filtration and crystallized from ethanol to give compound **13a-m**.

*2-Ethyl-4-hydroxy-2H-benzo[e][1,2]thiazine-3-carboxamide 1,1-dioxide (* ***13a*** *)*

Yield: 82%, mp: 242-245 °C; LC-MS (ESI): *m/z* 267 [M-H]^-^; IR (KBr): ν (cm^-1^) 3570, 3315 (NH_2_), 1653 (C=O); ^1^H NMR (400 MHz, DMSO-*d6*): *δ* 15.09 (broad s, 1 H, enolic OH), 8.19 (broad s, 2H, NH_2_), 7.99 (d, *J = *7.8 Hz, 1H, H_8_), 7.9-7.79 (m, 3H, H_5_, H_6_, H_7_), 3.41 (q, *J = *5.9 Hz, 2H, CH_2_-CH_3_), 0.64 (t, *J = *5.9 Hz, 3H CH_2_-CH_3_); ^13^C NMR (400 MHz, DMSO-*d6*) δ 171.75, 159.03, 138.02, 133.55, 133.17, 128.79, 126.53, 122.96, 107.80, 47.26, 10.61. Anal.Calcd. for C_11_H_12_N_2_O_4_S: C, 49.25; H, 4.51; N, 10.44. Found: C, 49.44; H, 4.69; N, 10.24.

*2-(Cyclohexylmethyl)-4-hydroxy-2H-benzo[e][1,2]thiazine-3-carboxamide 1,1-dioxide (* ***13b*** *)*

Yield: 87%, mp: 263-264 °C; LC-MS (ESI): *m/z* 335 [M-H]^-^; ]^-^; IR (KBr): ν (cm^-1^) 3307, 3178 (NH_2_), 1668 (C=O); ^1^H NMR (400 MHz, DMSO-*d6*): *δ* 14.79 (broad s, 1 H, enolic OH), 8.15 (broad s, 2H, NH_2_), 8-7.95 (m, 1H, H_8_), 7.89-7.76 (m, 3H, H_5_, H_6_, H_7_), 3.26 (d,* J = *6.0 Hz, 2H, cyclohexyl-CH_2_), 1.47-0.6 (m, 11 H, cyclohexyl); ^13^C NMR (400 MHz, DMSO-*d6*) δ 171.32, 157.99, 137.49, 133.42, 133.06, 128.60, 126.47, 122.98, 109.37, 57.75, 35.99, 31.56, 25.85, 25.63. Anal.Calcd. for C_16_H_20_N_2_O_4_S: C, 57.13; H, 5.99; N, 8.33. Found: C, 57.01; H, 6.20; N, 8.13.

*2-Benzyl-4-hydroxy-2H-benzo[e][1,2]thiazine-3-carboxamide 1,1-dioxide (* ***13c*** *)*

Yield: 72%, mp: 270-275 °C; LC-MS (ESI): *m/z* 329 [M-H]^-^; IR (KBr): ν (cm^-1^) 3252, 3175 (NH_2_), 1659 (C=O); ^1^H NMR (400 MHz, DMSO-*d6*): δ 14.99 (broad s, 1 H, enolic OH), 8.30 (broad s, 2H, NH_2_), 7.67 (d, *J = *7.6 Hz, 1H, H_8_), 7.63 (t, *J = *7.8 Hz, 1H, H_6_), 7.55 (t, *J = *7.8 Hz, 1H, H_7_), 7.45 (d, *J = *7.6 Hz, 1H, H_5_), 6.99-6.82 (m, 5H, benzyl), 4.9-3.8 (m, 2H, CH_2_); ^13^C NMR (400 MHz, DMSO-*d6*) δ 171.42, 159.79, 137.70, 132.80, 132.39, 131.72, 130.11, 128.53, 128.40, 127.75, 125.79, 122.85, 106.88, 55.09. Anal.Calcd. for C_16_H_14_N_2_O_4_S: C, 58.17; H, 4.27; N, 8.48. Found: C, 58.33; H, 4.51; N, 8.66.

*4-Hydroxy-2-(4-methylbenzyl)-2H-benzo[e][1,2]thiazine-3-carboxamide 1,1-dioxide (* ***13d*** *)*

Yield: 65%, mp: 276-278 °C; LC-MS (ESI): *m/z* 343 [M-H]^-^; IR (KBr): ν (cm^-1^) 3247, 3154 (NH_2_), 1667 (C=O); ^1^H NMR (400 MHz, DMSO-*d6*): δ 14.97 (broad s, 1 H, enolic OH), 8.26 (broad s, 2H, NH_2_), 7.7-7.6 (m, 2H, H_8 _& H_6_), 7.57 (t, *J = *8.0 Hz, 1H, H_7_), 7.46 (d, *J = *7.7 Hz, 1H, H_5_), 6.72-6.67 (m, 4H, 4-methylbenzyl), 4.85-4.1 (m, 2H, CH_2_), 2.50 (s, 3H, CH_3_); ^13^C NMR (400 MHz, DMSO-*d6*) δ 171.46, 159.79, 137.72, 137.65, 132.63, 132.37, 130.02, 128.82, 128.60, 128.23, 125.83, 122.89, 106.91, 54.88, 21.00. Anal.Calcd. for C_17_H_16_N_2_O_4_S: C, 59.29; H, 4.68; N, 8.13. Found: C, 59.40; H, 4.38; N, 7.95.

*4-Hydroxy-2-(2-methylbenzyl)-2H-benzo[e][1,2]thiazine-3-carboxamide 1,1-dioxide (* ***13e*** *)*

Yield: 73%, mp: 268-271 °C; LC-MS (ESI): *m/z* 343 [M-H]^-^; IR (KBr): ν (cm^-1^) 3236, 3146 (NH_2_), 1647 (C=O); ^1^H NMR (400 MHz, DMSO-*d6*): δ 14.92 (broad s, 1 H, enolic OH), 8.20 (broad s, 2H, NH_2_), 7.73 (d, *J = *7.8 Hz, 1H, H_8_), 7.67 (t, *J = *7.8 Hz, 1H, H_6_), 7.60 (t, *J = *7.8 Hz, 1H, H_7_), 7.49 (d, *J = *7.8 Hz, 1H, H_5_), 7.58-6.60 (m, 4H, 2-methylbenzyl), 4.59 (s, 2H, CH_2_), 2.1 (s, 3H, CH_3_); ^13^C NMR (400 MHz, DMSO-*d6*) δ 171.88, 159.59, 137.75, 137.43, 133, 132.50, 131.37, 130.65, 128.54, 125.87, 125.26, 123.10, 107.26, 51.94, 18.89. Anal.Calcd. for C_17_H_16_N_2_O_4_S: C, 59.29; H, 4.68; N, 8.13. Found: C, 59.45; H, 4.74; N, 8.30.

*4-Hydroxy-2-(3-methoxybenzyl)-2H-benzo[e][1,2]thiazine-3-carboxamide 1,1-dioxide (* ***13f*** *)*

Yield: 78%, mp: 273-275 °C; LC-MS (ESI): *m/z* 359 [M-H]^-^; IR (KBr): ν (cm^-1^) 3237, 3157 (NH_2_), 1667 (C=O), 1253 (C-O); ^1^H NMR (400 MHz, DMSO-*d6*): δ 15.00 (broad s, 1 H, enolic OH), 8.29 (broad s, 2H, NH_2_), 7.71 (d, *J = *7.0 Hz, 1H, H_8_), 7.64 (t, *J = *7.5 Hz, 1H, H_6_), 7.58 (t, *J = *7.2 Hz, 1H, H_7_), 7.5 (d, *J = *7.5 Hz, 1H, H_5_), 6.86-6.55 (m, 4H, 3-methoxybenzyl), 4.91-4.15 (m, 2H, CH_2_), 3.49 (s, 3H, OCH_3_); ^13^C NMR (400 MHz, DMSO-*d6*) δ 171.40, 159.77, 158.71, 137.74, 133.13, 132.80, 132.35, 128.86, 125.83, 122.87, 122.62, 115.05, 114.53, 106.99, 55.16, 55.11. Anal.Calcd. for C_17_H_16_N_2_O_5_S: C, 56.66; H, 4.48; N, 7.77. Found: C, 56.35; H, 4.28; N, 7.45.

*2-(2-Chlorobenzyl)-4-hydroxy-2H-benzo[e][1,2]thiazine-3-carboxamide 1,1-dioxide (* ***13g*** *)*

Yield: 76%, mp: 278-280 °C; LC-MS (ESI): *m/z* 363 [M-H]^-^; IR (KBr): ν (cm^-1^) 3218, 3175 (NH_2_), 1647 (C=O); ^1^H NMR (400 MHz, DMSO-*d6*): δ 15.80 (broad s, 1 H, enolic OH), 8.25 (broad s, 2H, NH_2_), 7.76 (d, *J = *7.2 Hz, 1H, H_8_), 7.66 (t, *J = *6.72 Hz, 1H, H_7_), 7.60 (t, *J = *7.7 Hz, 1H, H_6_), 7.48 (d, *J = *7.6 Hz, 1H, H_5_), 7.19 (d, *J = *7.7, 1H, 2-chlorobenzyl H_6_), 7.05-7.01 (m, 2H, 2-chlorobenzyl H_3 _& H_4_), 6.76-6.73 (t, *J = *7.3 Hz, 1H, 2-chlorobenzyl H_5_), 4.75 (s, 2H, CH_2_); ^13^C NMR (400 MHz, DMSO-*d6*) δ 171.92, 159.24, 137.84, 134.63, 133.03, 132.89, 132.42, 130.49, 129.80, 129.34, 128.48, 126.64, 125.77, 123.21, 107.15, 56.50. Anal.Calcd. for C_16_H_13_ClN_2_O_4_S: C, 52.68; H, 3.59; N, 7.68. Found: C, 52.39; H, 3.77; N, 7.80.

*2-(4-Chlorobenzyl)-4-hydroxy-2H-benzo[e][1,2]thiazine-3-carboxamide 1,1-dioxide (* ***13h*** *)*

 Yield: 68%, mp: 283-285 °C; LC-MS (ESI): *m/z* 363 [M-H]^-^; IR (KBr): ν (cm^-1^) 3209, 3137 (NH_2_), 1662 (C=O); ^1^H NMR (400 MHz, DMSO-*d6*): δ 15.05 (broad s, 1 H, enolic OH), 8.31 (broad s, 2H, NH_2_), 7.68-7.58 (m, 3H, H_6_, H_7_, H_8_), 7.50 (d, *J = *7.5 Hz, 1H, H_5_), 6.97 (d, *J = *8.4 Hz, 2H, 4-chlorobenzyl H_2_ & H_6_), 6.85 (d, *J = *8.4 Hz, 2H, 4-chlorobenzyl H_3_ & H_5_), 3.99 (s, 2H, CH_2_); ^13^C NMR (400 MHz, DMSO-*d6*) δ 171.38, 159.92, 137.60, 133.27, 132.84, 132.53, 131.93, 130.68, 128.50, 127.67, 125.89, 123.01, 106.83, 54.39. Anal.Calcd. for C_16_H_13_ClN_2_O_4_S: C, 52.68; H, 3.59; N, 7.68. Found: C, 52.85; H, 3.69; N, 7.48.

*2-(3,4-Dichlorobenzyl)-4-hydroxy-2H-benzo[e][1,2]thiazine-3-carboxamide 1,1-dioxide (* ***13i*** *)*

 Yield: 86%, mp: 291 °C (decomposed); LC-MS (ESI): *m/z* [M-H]^-^; IR (KBr): ν (cm^-1^) 3246, 3147 (NH_2_), 1658 (C=O); ^1^H NMR (400 MHz, DMSO-*d6*): δ 15.01 (broad s, 1 H, enolic OH), 8.30 (broad s, 2H, NH_2_), 7.7-7.6 (m, 3H, H_6_, H_7_, H_8_), 7.54 (d, *J = *7.3 Hz, 1H, H_5_), 7.21-6.85 (m, 3H, 3,4-dichlorobenzyl), 4.48 (s, 2H, CH_2_); ^13^C NMR (400 MHz, DMSO-*d6*) δ 171.33, 160.11, 137.64, 132.93, 132.69, 132.56, 132.08, 131.25, 130.64, 130.44, 129.80, 128.38, 125.90, 123.01, 106.82, 54.04. Anal.Calcd. for C_16_H_12_Cl_2_N_2_O_4_S: C, 48.14; H, 3.03; N, 7.02. Found: C, 48.33; H, 3.22; N, 7.20. 

*2-(2-Fluorobenzyl)-4-hydroxy-2H-benzo[e][1,2]thiazine-3-carboxamide 1,1-dioxide (* ***13j*** *)*

Yield: 71%, mp: 268-273 °C; LC-MS (ESI): *m/z* 347 [M-H]^-^; IR (KBr): ν (cm^-1^) 3228, 3153 (NH_2_), 1659 (C=O); ^1^H NMR (400 MHz, DMSO-*d6*): δ 15.05 (broad s, 1 H, enolic OH), 8.30 (broad s, 2H, NH_2_), 7.73 (d, *J = *7.2 Hz, 1H, H_8_), 7.65 (t, *J = *7.4 Hz, 1H, H_6_), 7.57 (t, *J = *7.4 Hz, 1H, H_7_), 7.1-7 (m, 1H, H_5_) 6.9-6.65 (m, 4H, 2-fluorobenzyl), 4.6 (s, 2H, CH_2_); ^13^C NMR (400 MHz, DMSO-*d6*) δ 171.58, 162.30, 159.85, 159.37, 137.47, 132.98, 132.59, 132.55, 132.37, 131.08, 130.99, 128.44, 125.67, 123.86, 123.83, 123.08, 119.05, 118.90, 115.27, 115.05, 107.11, 56.50. Anal.Calcd. for C_16_H_13_FN_2_O_4_S: C, 55.17; H, 3.76; N, 8.04. Found: C, 55.39; H, 3.99; N, 7.95.

*2-(3-Fluorobenzyl)-4-hydroxy-2H-benzo[e][1,2]thiazine-3-carboxamide 1,1-dioxide (* ***13k*** *)*

Yield: 73%, mp: 271-275 °C; LC-MS (ESI): *m/z* 347 [M-H]^-^; IR (KBr): ν (cm^-1^) 3238, 3148 (NH_2_), 1664 (C=O); ^1^H NMR (400 MHz, DMSO-*d6*): δ 15.04 (broad s, 1 H, enolic OH), 8.31 (broad s, 2H, NH_2_), 7.74-7.55 (m, 3H, H_6_, H_7_, H_8_), 7.51 (d, *J = *7.4 Hz, 1H, H_5_), 7.0 (dd, *J = *14.2, 7.7 Hz, 1H, 3-fluorobenzyl H_4_), 6.8 (t, *J = *8.3 Hz, 1H, 3-fluorobenzyl H_5_), 6.68 (d, *J = *7.6, 1H, 3-fluorobenzyl H_6_), 6.61 (d, *J = *9.5 Hz, 1H, 3-fluorobenzyl H_2_), 4.55 (s, 2H, CH_2_); ^13^C NMR (400 MHz, DMSO-*d6*) δ 171.34, 159.87, 137.67, 134.35, 133.00, 132.52, 129.81, 129.73, 128.45, 126.48, 125.87, 122.94, 116.89, 116.68, 115.37, 115.16, 106.88, 54.51. Anal.Calcd. for C_16_H_13_FN_2_O_4_S: C, 55.17; H, 3.76; N, 8.04. Found: C, 55.40; H, 3.56; N, 8.22.

*2-(4-Fluorobenzyl)-4-hydroxy-2H-benzo[e][1,2]thiazine-3-carboxamide 1,1-dioxide (* ***13l*** *)*

Yield: 76%, mp: 273-274 °C; LC-MS (ESI): *m/z* 347 [M-H]^-^; IR (KBr): ν (cm^-1^) 3238, 3138 (NH_2_), 1649 (C=O); ^1^H NMR (400 MHz, DMSO-*d6*): δ 15.06 (broad s, 1 H, enolic OH), 8.32 (broad s, 2H, NH_2_), 7.70-7.56 (m, 3H, H_6_, H_7_, H_8_), 7.51 (d, *J = *7.5 Hz, 1H, H_5_), 6.85 (d, *J = *8.3 Hz, 2H, 4-fluorobenzyl H_2 _& H_6_), 6.74 (t, *J = *8.3 Hz, 2H, 4-fluorobenzyl H_3 _& H_5_), 4.06 (s, 2H, CH_2_); ^13^C NMR (400 MHz, DMSO-*d6*) δ 171.39 ,163.33, 160.90, 159.94, 137.65, 132.84, 132.47, 132.28, 132.20, 128.55, 128.04, 128.01, 125.82, 122.94, 114.60, 114.39, 106.75, 54.33. Anal.Calcd. for C_16_H_13_FN_2_O_4_S: C, 55.17; H, 3.76; N, 8.04. Found: C, 55.37; H, 3.95; N, 8.14.

*4-Hydroxy-2-(4-nitrobenzyl)-2H-benzo[e][1,2]thiazine-3-carboxamide 1,1-dioxide (* ***13m*** *)*

Yield: 83%, mp: 288 °C (decomposed); LC-MS (ESI): *m/z* 374 [M-H]^-^; IR (KBr): ν (cm^-1^) 3237, 3157 (NH_2_), 1667 (C=O), 1343, 1558 (NO_2_); ^1^H NMR (400 MHz, DMSO-*d6*): δ = 15.09 (broad s, 1 H, enolic OH), 8.34 (broad s, 2H, NH_2_), 7.8 (d, *J = *8.6 Hz, 2H, 4-nitrobenzyl H_3_ & H_5_), 7.67-7.61 (m, 2H, H_6_ & H_8_), 7.55 (t, *J = *7.8 Hz, 1H, H_7_), 7.47 (d, *J = *7.8 Hz, 1H, H_5_), 7.15 (d, *J = *8.6 Hz, 2H, 4-nitrobenzyl H_2_ & H_6_) , 4.61 (s, 2H, CH_2_); ^13^C NMR (400 MHz, DMSO-*d6*) δ 171.30, 160.04, 147.31, 139.13, 137.45, 133.00, 132.66, 131.57, 128.36, 125.97, 123.14, 122.64, 1.6.87, 54.28. Anal.Calcd. for C_16_H_13_N_3_O_6_S: C, 51.20; H, 3.49; N, 11.20. Found: C, 51.40; H, 3.12; N, 11.32.

*Molecular docking study*

Molecular modeling was performed using the Autodock Vina (34). 3OYA was used for binding mode analysis of HIV IN inhibitory activity. The protein and ligands were prepared in Autodock tools 1.5.6 from the MGL Tools package (35). The co-crystallized ligand and water molecules were extracted, Kollman charges were added, nonpolar hydrogens were merged, and AutoDock4 atom type was assigned to the protein structure. The ligand was created and minimized using HyperChem 8.0 (36). The active site was defined as a Grid box around the crystallographic ligand raltegravir in 20 × 20 × 20 dimensions. The molecule was docked in the active site, and the bioactive conformations were generated using Autodock Vina.

*In-vitro anti-HIV-1 and cytotoxicity assay method*

The anti-HIV-1 activity of target compounds was evaluated in a single cycle replication assay evaluated in our laboratory and reported previously. In this assay, the single-cycle replicable HIV NL4-3 virions (200 ng p24) were inoculated concurrently with compounds in different concentrations to the Hela cells. The inhibition rate (%) of p24 expression was measured by capture ELISA (Biomerieux, France) 72 hours after inoculation. Percentage inhibition of p24 expression in treated culture was calculated as inhibition rate of p24 (%). The XTT (sodium3-[1(phenylaminocarbonyl)-3,4-tetrazolium]-bis(4-methoxy-6-nitro)benzene sulfonic acid) proliferation assay was conducted to evaluate the cellular toxicity according to the kit instructions. The cytotoxic concentration that reduced the number of viable cells by 50% (CC_50_) was calculated after the determination of p24 load in the HIV-1 replication assay plates (37-40).

## Results and Discussion

*Chemistry*

**Scheme 1:**
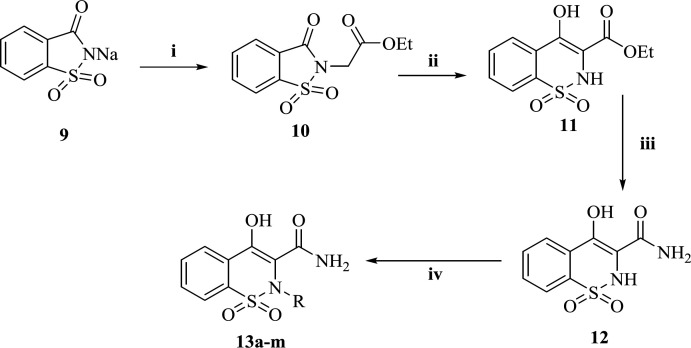
reagents and conditions: (i) Ethyl chloroacetate, DMF, 120 °C, 3 h, (ii) Na, Dry ethanol, reflux, 2 h, (iii) Ammonia, water, r.t, overnight. (iv) alkyl or benzyl halide derivatives, DMF, K2CO3, r.t, 1.5-2 h

The general pathway for the preparation of final compounds was shown in Scheme 1. Commercially available saccharin (**9**) underwent N-alkylation in DMF with ethyl chloroacetate to afford compound **10**. In a well-known reaction called Gabriel-Colman Rearrangement, compound **10**, under highly basic conditions, was converted to ethyl 4-hydroxy-2*H*-benzo[*e*][1,2]thiazine-3-carboxylate 1,1-dioxide (**11**). Then, ester (**11**) was reacted with ammonia to give 4-hydroxy-2*H*-benzo[*e*][1,2]thiazine-3-carboxamide 1,1-dioxide (**12**). Final compounds (**13a-m**) were achieved by reaction of compound **12 **with alkyl or benzyl halide derivatives. All the synthesized derivatives were well characterized with IR, ^1^H-NMR, ^13^C-NMR spectroscopy and LC-MS technique.

*Biological evaluation*

Fourteen 1,2-benzothiazine-3-carboxamide 1,1-dioxide derivatives containing different substituents at N-2 positions (**12** and **13a-m**) were synthesized and evaluated to inhibit the single-cycle HIV replication inhibition HeLa cell culture. Cytotoxicity of compounds was also tested in the same cell line to ensure the safety of compounds. Raltegravir was used as a positive control. Anti-HIV activity and cytotoxicity of compounds were expressed as EC_50_ and CC_50_, respectively. Selectivity Index (SI) was calculated using CC_50_/EC_50_ ratio. Biological evaluation results are summarized in Table 1.

All target compounds showed no considerable cytotoxicity with CC_50_ values > 500 mM (except **13b**), rendering them suitable as a new scaffold for anti-HIV drug development. A small aliphatic group like ethyl, bulkier groups like cyclohexylmethyl, benzyl and mono- and di-substituted benzyl groups were introduced to study the structure-activity relationship. As shown in Table 1, target compounds exhibited EC_50_ values ranging from 20-110 mM that were better than the value previously reported for piroxicam (EC_50_ =120 mM). Most of the substituted derivatives showed higher potency than unsubstituted derivative **12** (EC_50_ = 105 mM). This suggested that the introduction of a hydrophobic group at the N-2 position is crucial for anti-HIV activity. Among substituted derivatives **13a-m**, compounds with benzyl or mono-substituted benzyl groups (compounds **13c-h** and **13j-m**) were more active than compounds containing aliphatic groups (compounds **13a **and **13b**). Compound **13i** having a 3,5-dichlorobenzyl group showed moderate activity with an EC_50 _value of 110 µM. It was observed that substituents at the *para* position of benzyl ring provided higher potency when compared to *ortho* and *meta *positions. Among different electron-donating and electron-withdrawing groups applied as a substituent on benzyl ring, fluoro, nitro and methyl appeared to considerably benefit anti-HIV activity as compounds **13d**, **13l,** and **13m** exhibited the highest potency with EC_50 _values ranging from 20-25 µM. All these SAR observations indicated that a mixture of steric, electrostatic and hydrophobic effects should be blended to achieve the best anti-HIV activity.

In general, anti-HIV assay results revealed that modification of the central scaffold of piroxicam to increase antiviral potency was promising. Introduction a flexible 4-substituted benzyl portion on 2-position of the scaffold lead to significant improvement in anti-HIV activity. Compound **13l** containing 4-fluorobenzyl group was the most potent compound with an EC_50_ value of 20 µM and an encouraging SI value of 32.5. These results conform to the common pharmacophore of IN inhibitors in which a fluorobenzyl group associated with a chelating moiety is required for the best inhibitory activity.

*Molecular docking study*

**Figure 3:**
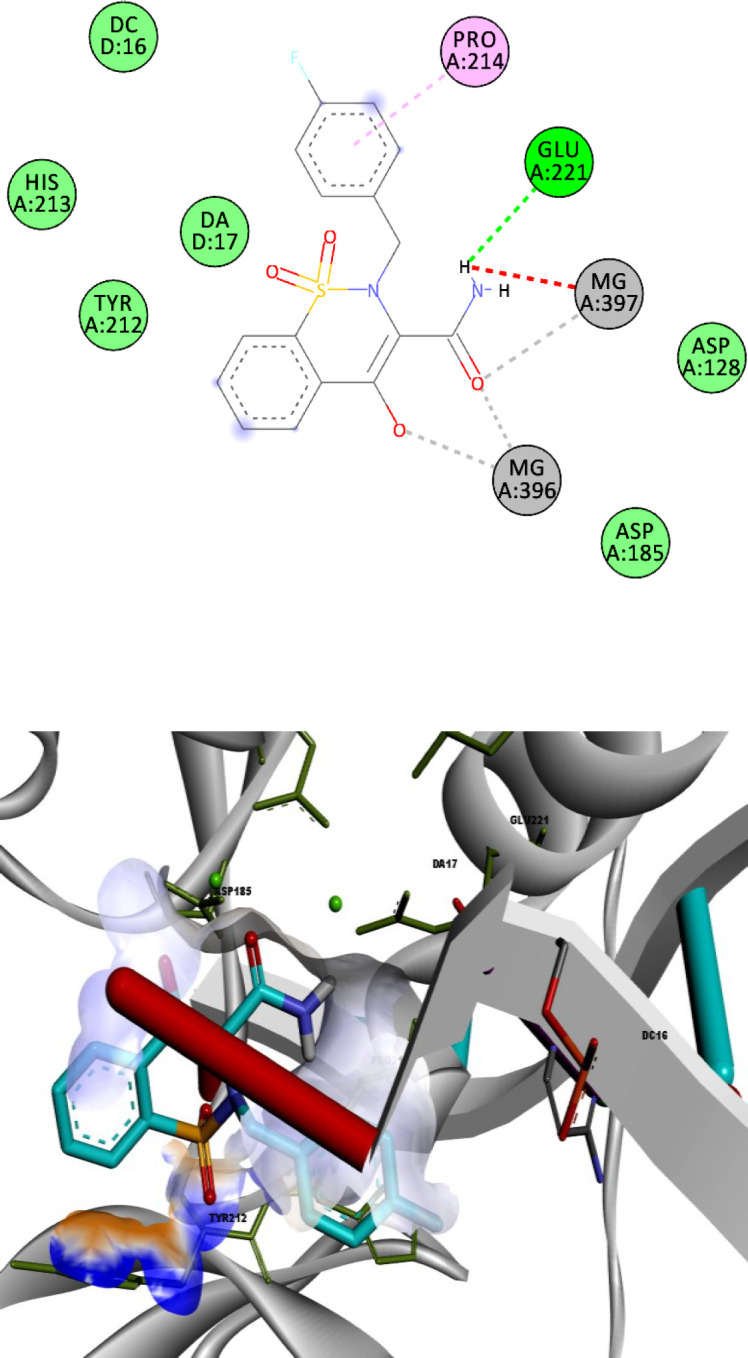
2D and 3D alignment of best-docked conformer of compound **13l** (shown in blue) in the PFV IN active site

To define the possible mechanism of action of target compounds, a molecular docking study to integrase active site was conducted. The molecular docking study was performed with Autodock vina, and Discovery Studio Visualizer2017 was used for visualization and analysis. The Prototype Foamy Virus integrase (PFV-IN) structure in complex with two Mg^2+^ ions and a double chain DNA at 2.65 Å resolution (PDB:3OYA) was used as the receptor for docking study (41, 42). To validate the docking study, the co-crystalized ligand raltegravir, was also docked under the same condition and superimposed on co-crystalized ligand pose (RMSD = 0.001) and displayed high binding affinity (-12.8 kcal/mol). Docking results indicated that all docked compounds occupied the same place in the integrase active site with an affinity binding energy range from -7.5 to -9.6 kcal/mol (Table 1). According to docking outcomes, there was a correlation between affinity binding energies and the anti-HIV activity of compounds. Compounds possessing benzyl or mono-substituted benzyl groups (**13c-m**) showed higher binding affinity than compounds containing aliphatic groups (**13a** and **13b**) or unsubstituted compound **12**. Compound **13l**, the most active molecule in the anti-HIV assay, showed the highest binding affinity (-9.6 kcal/mol). The 3D and 2D alignment of compound **13l** in the active site was shown in Figure 3. As expected, hydroxy and carboxamide groups of compound **13l** were involved in the metal acceptor interactions with the Mg^2+^ ions in 2.74, 2.22, 2.55 and 2.66 Å distances. The 4-fluorobenzyl moiety was inserted into the protein-DNA interfacial hydrophobic pocket. The 4-fluorobenzyl moiety also formed additional hydrophobic interaction with the Pro214 residue. In addition, the central 1,2-benzothiazine 1,1-dioxide ring was able to participate in π-stacking bonding with the terminal 3′-deoxyadenosine A17 (DA17).

**Figure 4:**
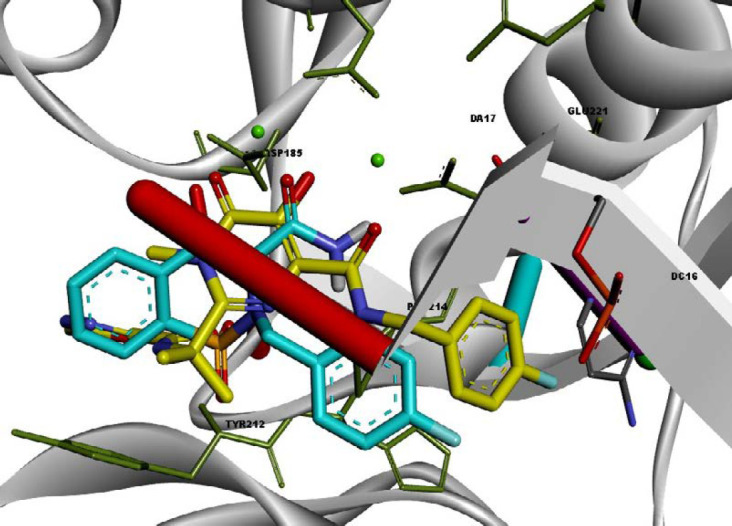
Superimposition of compound **13l** (shown in blue) on raltegravir (shown in yellow) in the PFV IN active site

A structure overlay of the compound **13l **on raltegravir displayed a similar orientation for the chelating moieties of both **13l** and raltegravir within the IN active site (Figure 4). The 4-fluorobenzyl moiety of compound **13l** oriented towards the hydrophobic pocket but not as deeply as the raltegravir one. This may be the reason for the lower potency of **13l** in respect to raltegravir. Collectively, the binding pose of the tested compounds was similar to HIV IN inhibitors, suggesting that their anti-HIV activity may be the result of IN inhibition.

**Table 1:** Bioassay data and docking score of compounds **12 **and** 13a–m**

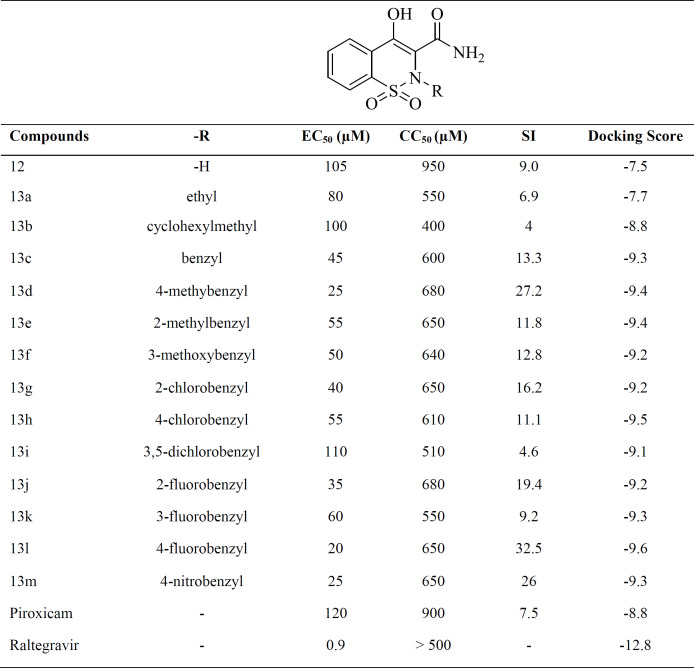

## Conclusion

A novel series of piroxicam analogs were designed based on the features required for HIV IN inhibition. Cell-based anti-HIV assay indicated that most of the tested compounds possess promising activity associated with no significant cytotoxicity. Among them, compounds **13d**, **13l** and **13m** exhibited the highest potency with EC_50_ values ranging from 20-25 µM and SI > 26. Docking studies showed that the compounds interact with IN in a similar mode to co-crystalized raltegravir. These findings prompted us to focus on further modifications of the piroxicam scaffold to increase antiviral activity. 
